# Granger Causality Analysis of Chignolin Folding

**DOI:** 10.1021/acs.jctc.1c00945

**Published:** 2022-02-15

**Authors:** Marcin Sobieraj, Piotr Setny

**Affiliations:** † Faculty of Physics, University of Warsaw, Pasteura 5, 02-093 Warsaw, Poland; ‡ Centre of New Technologies, University of Warsaw, Banacha 2c, 02-097 Warsaw, Poland

## Abstract

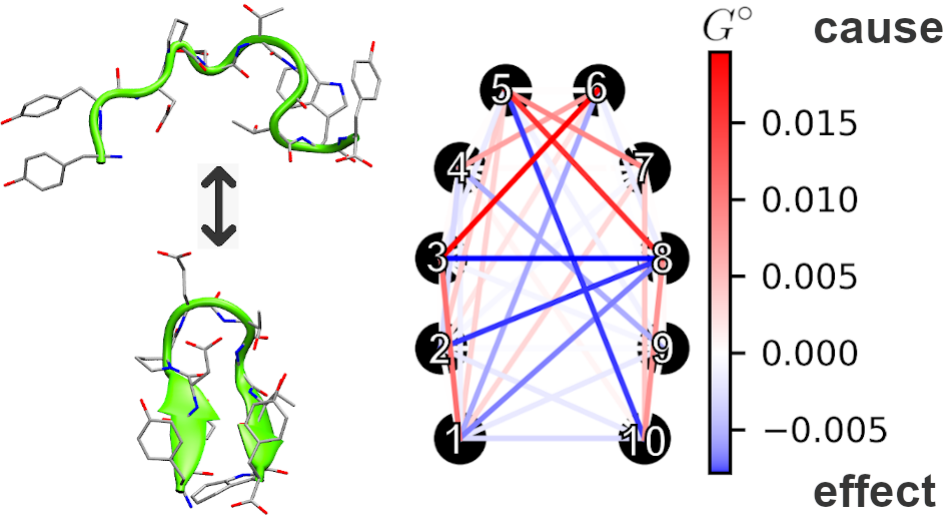

Constantly advancing computer simulations of biomolecules provide huge amounts of data that are difficult to interpret. In particular, obtaining insights into functional aspects of macromolecular dynamics, often related to cascades of transient events, calls for methodologies that depart from the well-grounded framework of equilibrium statistical physics. One of the approaches toward the analysis of complex temporal data which has found applications in the fields of neuroscience and econometrics is Granger causality analysis. It allows determining which components of multidimensional time series are most influential for the evolution of the entire system, thus providing insights into causal relations within the dynamic structure of interest. In this work, we apply Granger analysis to a long molecular dynamics trajectory depicting repetitive folding and unfolding of a mini β-hairpin protein, CLN025. We find objective, quantitative evidence indicating that rearrangements within the hairpin turn region are determinant for protein folding and unfolding. On the contrary, interactions between hairpin arms score low on the causality scale. Taken together, these findings clearly favor the concept of zipperlike folding, which is one of two postulated β-hairpin folding mechanisms. More importantly, the results demonstrate the possibility of a conclusive application of Granger causality analysis to a biomolecular system.

## 1 Introduction

Molecular dynamics (MD) simulations provide increasingly comprehensive insights into the functioning of biomolecular systems.^1−3^ One prominent area which has been fruitfully explored by means of MD is the problem of protein folding.^4−7^ Numerous studies have demonstrated that polypeptide chains described by atomistic force fields can successfully reach experimentally determined native states guided solely by sequence-based effects.^8−11^ Although less powerful in terms of practical ability to deliver sequence-based structure predictions compared to specialized approaches, in particular those based on extremely successful application of machine learning techniques,^12^ MD simulations are unique in that they provide means to trace and, possibly, explain the details of the actual folding process. Still, even having access to atomistic, time-resolved folding trajectories does not always ensure unambiguous, objective interpretation of events that occur along the folding pathway or a clear understanding of the underlying biophysical driving forces.^13−15^

In this respect, even the folding of short β-hairpin structures is far from being clear.^16,17^ Regarded as minimalistic protein models for their ability to achieve well-defined native states while having as few as 10 amino acids, they have been extensively investigated.^18−23^ So far, two major theories concerning the sequence of events have been formulated. The first one assumes that the folding pathway starts with the appearance of a turn in the middle of the polypeptide chain and advances by outward propagation of hairpin contacts in a zipperlike manner.^24^ The second one postulates the collapse of hydrophobic residues within hairpin arms as the primary event, followed by series of structural rearrangements that lead to turn stabilization and the formation of interstrand contacts.^25,26^ Notably, both views received support from experimental and computational studies of a few β-hairpin structures, which possibly implies that in fact there is no single, uniform mechanism of β-hairpin formation.

Particularly well-suited systems for computer-based investigation of hairpin folding are a human-designed miniprotein called chignolin^27^ and its later variant CLN025.^28^ Both comprise only 10 amino acids, form stable hairpin structures amenable for nuclear magnetic resonance and X-ray crystallography, and, with experimentally confirmed folding times of only a few hundred nanoseconds, can be exhaustively sampled by fully atomistic simulations.^29−31^ Accordingly, a number of studies have addressed chignolin and CLN025 reversible folding in an explicit aqueous solvent, finding support for both the zipperlike^29,32−34^ and the hydrophobic collapse driven^35^ mechanisms, but also suggesting variability^9^ and possible force field dependence of available pathways.^31,36^

Certainly the lack of consensus concerning chignolin and CLN025 folding, at least to some extent, stems from discrepancies between force field models. Whereas the native state is generally well captured by all major models, only very subtle differences are enough to change the properties of the unfolded ensemble^37^ as well as the nature and sequence of intermediate states, as has been demonstrated in a recent study.^31^ In addition to that, however, there exist only limited analysis options providing insights into detailed temporal characteristics of biomolecular structure rearrangements. Major efforts in this respect have been devoted to the development of Markov state models (MSMs) and associated methodologies for objective determination of relevant system representation.^38^ The resulting framework allows assembling information from multiple, relatively short simulations into a complete kinetic model which can then be used to characterize significantly longer relaxation processes and their associated structural changes. While extremely powerful in determining pathways interconnecting meaningful system states and associated time scales, MSMs do not reveal, however, the significance of temporal relations between the occurring events. As a result, even having captured the kinetically relevant reaction coordinate, it is still difficult to determine which elements of system dynamics are of importance for its propagation along the path.

A possible way to draw conclusions about temporal relations in complex processes is provided by Granger causality analysis (GC). It was first proposed in 1969^39^ and has found applications predominantly in economics, finance, and neuroscience.^40−45^ Given a process described by multidimensional time series, GC determines whether, based on the knowledge of one time series, *X*, it is possible to probabilistically predict the behavior of another time series, *Y*. Causality considered in this way, expressed in the following as *X* Granger-causes (G-causes) *Y*, avoids the deeply philosophical question of the “true cause” of a given phenomenon and, more importantly, provides an effective statistical procedure for measuring the strength of temporal relationships. The original formulation of GC was founded on the framework of multivariate autoregressive models, thus relying on the existence of linear couplings within the system. The general idea was further extended to include nonlinear effects by means of information theory, employing transfer entropy^46^ instead of time-shifted correlation to measure temporal dependencies between data channels.^47^ Both formalisms have been applied to the analysis of molecular dynamics simulations utilizing source data in the form of time series describing fluctuations of atomic positions in Cartesian space,^48−50^ residue-based fraction of native contacts,^51^ or custom molecular descriptors.^52,53^ Most recently, causal relations have been inferred from a transfer entropy measure which, instead of directly using time-resolved signals, operated on probability distributions involving elements of transition matrices representing local Markov state models, constructed for disjoined regions of a protein structure.^54^

In this article, we perform GC analysis of CLN025 folding based on a 106 μs long atomistic simulation performed by Lindorff-Larsen et al.^9^ The resulting trajectory contains multiple folding and unfolding events allowing for parametrization of a converged GC model. Instead of focusing on individual residues, we consider inter-residue distances, as putative causal relations between them can be interpreted in terms of dependences between the formation and disruption of particular physical interactions in the course of the (un)folding process. We demonstrate that without any preliminary knowledge-based assumptions the model clearly favors one of the debated chignolin folding mechanisms and points to the importance of transient structural motifs in the unfolded ensemble for subsequent steps toward the native state.

## 2 Methods

### 2.1 Multivariate Autoregressive Model

Multivariate autoregressive models (MVARs) are used to describe and analyze multidimensional temporal signals exploiting the existence of time-shifted correlations between individual components. In particular, they can be applied to forecast signal evolution based on linear combination of past values in respective channels. Given a multidimensional time series **x**(*t*) = {*x*_1_(*t*), ..., *x*_*K*_(*t*)} propagated with a time step Δ*t*, a prediction, **x**_pred_(*t*), of signal value at time *t* can be attempted based on *P* preceding steps in the following manner:
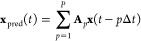
1

Here, **A**_*p*_ are *K* × *K* real matrices containing parameters defining an MVAR model of order *P*, which are determined under the assumption that the residual difference between real and predicted signals, Δ**x**(*t*) = **x**(*t*) – **x**_pred_(*t*), remains a white noise with stationary variance.

One of the possible ways to estimate model parameters is provided by the Yule–Walker method.^55,56^ Having obtained matrices of mixed second statistical moments of signal components **Γ**(*r*) = **Γ**^T^(−*r*) = ⟨**x**(*t*) **x**^T^(*t* – *r*Δ*t*)⟩ for *r* ∈ {0, ..., *P*}, the parameters are calculated by solving the following set of *P* + 1 linear equations (see the Supporting Information for practical details):
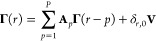
2where **V** = ⟨Δ**x**(*t*) Δ**x**^T^(*t*)⟩ is the white noise covariance matrix and δ_*r*,0_ is the Kronecker delta function.

### 2.2 Granger Causality

The elements of the residual white noise covariance matrix, **V**, in a parametrized MVAR model (eq 1) can be considered as quality indicators of model predictions for their respective signal components, based on data contained in preceding steps, in all signal channels. The Granger measure for a causal relation between two channels *i* and *j* is based on the assessment of how much the prediction error for the component *X*_*i*_ increases once the component *X*_*j*_ is excluded from model parametrization. Formally, the Granger causality from channel *j* to *i*, denoted as *J*_*j*→*i*_, is expressed as
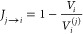
3where *V*_*i*_ and *V*_*i*_^(*j*)^ are *i*th diagonal elements of covariance matrices **V** and **V**^(*j*)^, obtained for the MVAR model parametrized with all the channels and excluding the *j*th channel, respectively. *J*_*j*→*i*_ constitute elements of the generally asymmetric Granger causality matrix, **J**, and can vary between 0 and 1. *J*_*j*→*i*_ = 0 indicates no G-causal relationship between given signal components, meaning that the omission of the *j*th channel does not affect the model’s ability to predict the *i*th channel. In turn, *J*_*j*→*i*_ = 1 reveals full coupling, implying that the model loses its ability to predict the *i*th channel, if the *j*th channel is excluded from parametrization.

### 2.3 Simulation Data and Processing Methods

The source trajectory representing 106 μs of the CLN025 β-hairpin fully atomistic molecular dynamics simulation in explicit solvent was obtained on request from D. E. Shaw Research.^9^ The trajectory comprised 534 743 frames saved with a time step of 0.2 ns. The peptide sequence Tyr-Tyr-Asp-Pro-Glu-Thr-Gly-Thr-Trp-Tyr was parametrized with the CHARMM22* force field, and the TIP3P model was used for water. Unassisted, spontaneous folding into a stable structure in good agreement with the crystallographic geometry (0.1 nm of Cα root-mean-square deviation, RMSD) was observed. A simulation temperature of 340 K was chosen to obtain multiple, reversible (un)folding events. Technical details of the simulation are given in the original report.^9^

In order to select a representative folded structure, the subset of trajectory frames spaced every 20 ns was clustered according to an all-atom RMSD using the Gromos algorithm as implemented in the Gromacs package, with a cutoff of 0.3 nm, and the reference geometry was determined as the central structure of the largest cluster. Subsequently, in order to determine a continuous reaction coordinate, ξ, for the (un)folding process, the all-atom RMSD with respect to this structure was used together with sines and cosines of peptide backbone ϕ and ψ angles as components in time independent component analysis (TICA).^57,58^ The TICA analysis in the resulting 37 dimensions was conducted with the kinetic mapping weighting scheme and a lag time of 120 ns, and its dominant independent component (IC) was used as a reaction coordinate. The choice of particular parameters was based on the method proposed by Best and Hummer,^59^ which provides objective criteria to optimize a reaction coordinate to properly capture reactive trajectories between stable states of interest (see Results and Discussion). To this end, we considered a number of trial reaction coordinates and selected the best among them (see the Supporting Information for details). We note that GC analysis itself does not require the definition of any reaction coordinate; however, it is useful to do so for the interpretation of results.

The potential of mean force (PMF) as a function of ξ was obtained based on the probability, *p*(ξ), of finding the trajectory at ξ, assuming PMF(ξ) = – ln *p*(ξ) + *F*_0_, with *F*_0_ constant and chosen such that the global minimum was 0. The PMF uncertainty is reported as plus or minus one standard deviation, based on calculations for the original trajectory split into five consecutive blocks.

To characterize intermediate steps and their representative structures, illustrating the (un)folding process, the ξ region between free energy minima corresponding to the folded, F, and the unfolded, U, states was split using six equidistant centers, and each trajectory frame was assigned to its closest center. Representative structures for such defined folding steps were determined as medoids of respective sets of frames, using distances in kinetically weighted, 37-dimensional TICA space.

For the calculation of the transition matrix and commitor function (CF), the trajectory was converted into an integer sequence based on discrete state numbers assigned to each frame according to the procedure described above, and it was processed using a median filter of 1 ns width. The CF was calculated as a probability that, upon leaving the state of interest, the trajectory reaches the F state prior to visiting the U state. Uncertainty of CF estimation at each point is reported as a range between minimal and maximal values obtained in independent calculations for the trajectory split into five consecutive blocks. The calculations of TICA, CF, and the transition matrix and their visualization were performed with the PyEMMA Python package.^60^

For the calculation of conditional probability, *p*(TP|ξ), of the system being on a transition path (TP) between F and U states at a given ξ,^59^ TPs were identified as continuous trajectory fragments connecting those two states in either direction, with no recrossing. The reaction coordinate region between F and U states was discretized into 50 bins, ξ_*i*_, and numbers of frames in each bin, *N*_*i*_ and *N*_*i*_^TP^, were recorded for the entire trajectory, as well as for TP fragments only, respectively, and were used to estimate *p*(TP|ξ_*i*_) = *N*_*i*_^TP^/*N*_*i*_. An error of the estimate is reported as plus or minus one standard deviation obtained for independent calculations involving *N*_*i*_, and *N*_*i*_^TP^ evaluation for five consecutive trajectory blocks.

Inter-residue distances were calculated as the shortest separation between the respective heavy atom sets. Hydrogen bonds were defined on the basis of geometric criteria involving an acceptor–donor distance of ≤0.32 nm and an acceptor–hydrogen donor angle in the range [130, 180] deg.^61^

The overlap, Ω(ξ) ∈ [0, 1], between the distribution of inter-residue distances at a given ξ value, discretized in 21 bins between F and U free energy minima, and their ensemble at state F (corresponding to the bin centered on the global PMF minimum) was calculated as an overlap area of two normal distributions of respective mean values and standard deviations using an overlap function implemented in the Python statistics library. It was further normalized such that the range of its variation along the transition path spanned the entire range between 0 and 1.

### 2.4 Granger Causality Analysis

To provide useful data for Granger analysis, the original molecular dynamics trajectory was featurized into a set of all 45 inter-residue distances, as defined above. The trajectory was split into two consecutive, equal parts, and each of them was processed independently to assess the convergence of results. Prior to performing the Granger analysis, the distances were normalized to have 0 mean and variance 1. MVAR parametrization and residual covariance matrix evaluation was carried out by using the Yule–Walker method as implemented in the Time Series 1.4 package of Mathematica 7.0. The optimal order, *P*, of the MVAR model was determined on the basis of the Schwarz–Bayes criterion,^62^ which seeks to minimize the following expression:
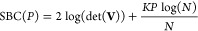
4with *N* being the number of time steps considered. Here, the first term on the right-hand side accounts for possibly accurate model predictions, while the second term penalizes model complexity. In our case, SBC(*P*) was stable with growing *P*, so we adopted *P* = 1. The low order of the resulting optimal MVAR model may be related to the relatively long trajectory time step.

In order to obtain insights into the involvement of particular contacts in G-causal relations, rather than to consider dependencies between all 990 possible contact pairs, we introduced the following measures:

- *G*_*i*_^←^ = ∑_*k*_*J*_*k*→*i*_, called *predictability to*, indicating the extent to which the behavior of the *i*th contact can be predicted based on the evolution of all remaining contacts
- *G*_*i*_^→^ = ∑_*k*_*J*_*I*→*k*_, called *predictability from*, indicating the extent to which information encoded by the evolution of the *i*th contact is useful for the prediction of all remaining contacts
(*G*_*i*_^→^ − *G*_*i*_^←^), called *origin predictability*, indicating whether a contact is a source, or an initiator, of events (*G*_*i*_^°^ > 0), or rather a sink, or a terminator, of events (*G*_*i*_^°^ < 0)
(*G*_*i*_^→^ + *G*_*i*_^←^), called *predictability indicator*, indicating the extent of general participance in G-causal relations

## 3 Results and Discussion

### 3.1 Folding Pathway

Even though GC analysis itself does not depend on prior determination of representative states or processes within the system of interest, their knowledge is essential for meaningful interpretation of the results. Thus, in order to capture relevant intermediate configurations of CLN025 (un)folding, we first devised a reaction coordinate, ξ, that would possibly well follow transition paths, TPs. To this end, we considered a number of descriptors characterizing peptide structure and validated their suitability to serve as a reaction coordinate using the method proposed by Best and Hummer.^59^ Briefly, the approach is based on the estimation of conditional probability that the system is on a TP, given its particular position along the reaction coordinate, *p*(TP|ξ). The extent to which the maximum of the resulting probability profile is able to reach the theoretical limit of 0.5 is then used to gauge the quality of the underlying reaction coordinate.

**Figure 1:**
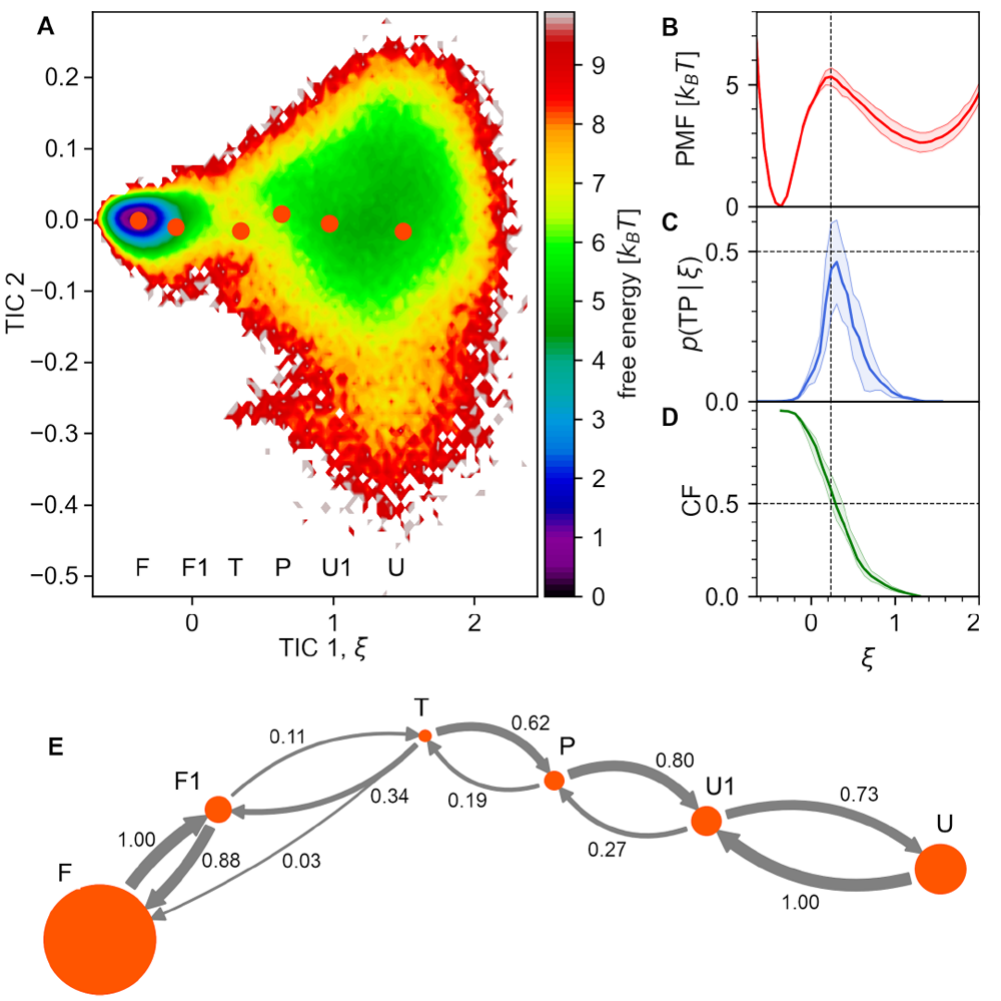
(A) Free energy map as a function of two dominant TICA ICs. Red dots indicate the location of representative frames for six (un)folding steps. (B–D) Descriptors of the folding process as functions of the reaction coordinate. Shaded areas indicate estimation errors (see text for details). (E) Transition matrix between six folding steps, with circle areas proportional to corresponding populations of simulation frames (transitions with probability < 0.03 not shown).

From various considered candidates for ξ (see the Supporting Information for details), we chose a combination of heavy atom RMSD from the representative native state structure with sines and cosines of backbone dihedral angles, transformed with the use of TICA.^58^ A two-dimensional free energy map as a function of the two first ICs of the TICA solution with a lag time of 120 ns (Figure 1A) indicates two unique minima corresponding to the folded and the unfolded states. The minimum free energy path between them leads predominantly along the first IC, and it was adopted as a one-dimensional reaction coordinate. The resulting PMF (Figure 1B) indicates folding free energy in the range of −2.5*k*_B_*T*, in agreement with previous studies based on the same trajectory.^9,31^ The height of the free energy barrier for unfolding, ∼5*k*_B_*T*, is, however, higher by ∼1*k*_*B*_*T* than that reported by Lindorff-Larsen in the original study.^9^ We attribute this difference to the possibility that the originally considered reaction coordinate based on the fraction of native contacts, *Q*, may not provide optimal resolution for the case of 10 residues only miniprotein, likely enriching the transition region with configurations that, in fact, do not belong to reactive trajectories. Indeed, whereas the maximum in *p*(TP|ξ) obtained for our proposed reaction coordinate is close to 0.5 (Figure 1C), this is not the case for our trial reaction coordinate based on *Q* (see the Supporting Information for details), in line with similar result reported for the same trajectory.^63^

In order to obtain representative structures of CLN025 along its (un)folding paths that would aid in the interpretation of GC results, we chose to select a set of states that (a) covers the reaction coordinate between the free energy minima for folded and unfolded states, (b) captures a geometry typical for the transition state region, and (c) is sufficiently small to enable visual analysis. We note that, as opposed to models aimed at the analysis of system kinetics, it is not necessary that the sets of states and related transition probabilities pass the Chapman–Kolmogorov test.^64^ Instead, as we seek to interpret putative causal relations in continuous processes such as (un)folding, it is desirable to obtain a set of steps that occur sequentially one by one during reactive trajectories. Given the above, we arbitrarily partitioned the reaction coordinate into six bins with centers evenly spaced between free energy minima and determined their representative structures as medoids in TICA space (Figure 1A). The resulting steps along the (un)folding pathway characterize folded (F), close to folded (F1), transition (T), preliminary (P), close to unfolded (U1), and unfolded (U) states. Assuming that hairpin folding is a process that leaves state U and reaches state F prior to coming back, we found in total 46 such folding events in the entire trajectory, with a mean duration of 9 ± 1 ns. In turn, the average duration of 46 unfolding processes was 14 ± 3 ns.

The relevance of the adopted reaction coordinate and chosen discrete states is further supported by the fact that the value of the reaction coordinate at which the CF calculated directly from trajectory passes the value of 0.5 (Figure 1D) is consistent with the location of the transition state suggested by the PMF maximum (Figure 1A). In addition, as evidenced by inspection of the accompanying transition matrix (Figure 1E), jumps between nonadjacent states are infrequent, with the highest rate of 0.03 observed for the transition from T directly to F, bypassing F1. This implies that the majority of (un)folding events indeed proceeds by visiting all consecutive steps.

### 3.2 Structural Characterization of Folding Steps

**Figure 2:**
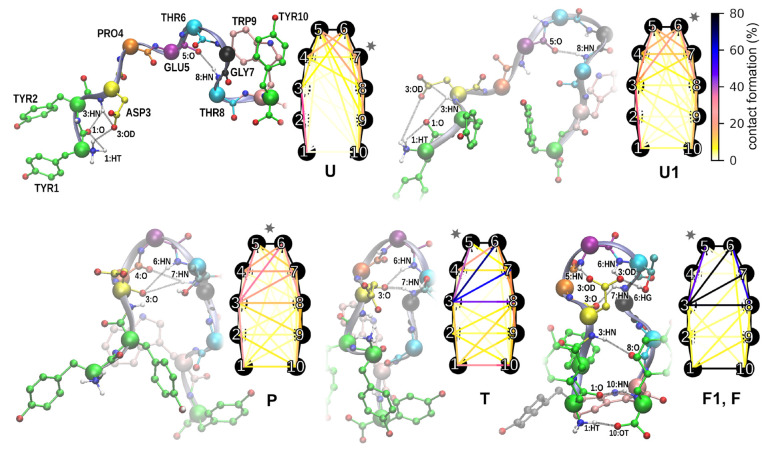
Representative structures for subsequent folding steps and schematic depiction of inter-residue contact frequencies. Shown are major hydrogen bonds captured in particular structures. Gray stars indicate turn locations in polypeptide backbone. Detailed frequencies of contact formation and the distribution of turn angles are provided in the Supporting Information.

**Figure 3:**
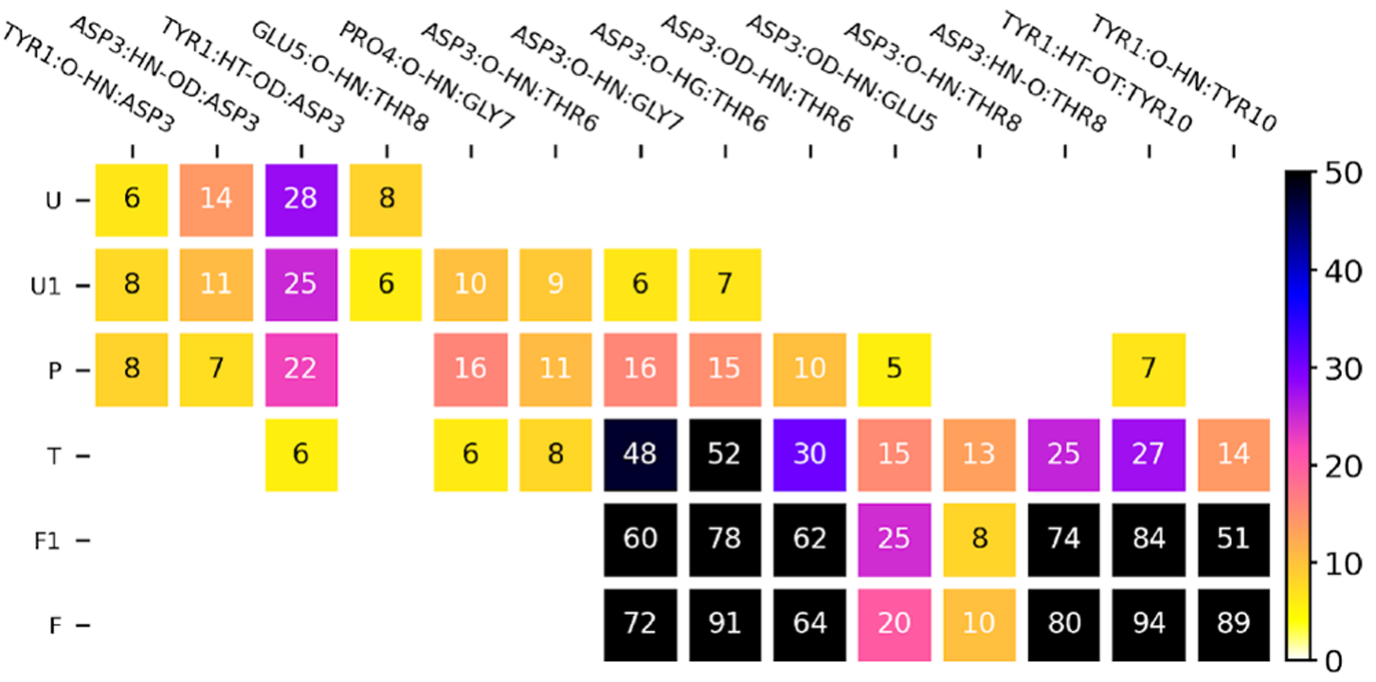
Fractions of hydrogen bonds formed in subsequent CLN025 (un)folding steps. Bonds formed in <5% of respective simulation frames are not shown.

Structural rearrangements between subsequent folding steps are analyzed on the basis of a set of representative geometries together with corresponding fractions of inter-residue contacts (Figure 2) as well as the most stable hydrogen bonds (Figure 3). As can be expected for a short peptide, the ensemble of unfolded structures is rather wide with practically no trace of the native geometry. Notably, though, there are two structural elements that are unique for the unfolded state (Figure 2U). The first one is a network of temporary hydrogen bonds at the N-terminus between Tyr1 and Asp3 that engages the aspartic acid side chain and pulls it away from its nativelike configuration. The second is a hydrogen bond between Glu5 and Thr8, which is responsible for the stabilization of a shallow turn in the region of residue 7, which is displaced with respect to the native hairpin turn located around residue 5.

In the part of the unfolded ensemble that is closer to the transition state (Figure 2U1), the dominant turn region shifts to residue 6, being supported by increasingly frequent main chain hydrogen bonds between Pro4 and Gly7, additionally augmented by new interactions between the Asp3 main chain oxygen atom and Thr6 and Gly7 amide nitrogen atoms.

This repositioning of the turn region toward residue 5 is continued in the preliminary step (Figure 2P), in which the interaction between Glu5 and Thr8 disappears in favor of further enhanced hydrogen bonds between Pro4 and Gly7 as well as the Asp3 main chain with Thr6 and Gly7. Notably, the prevalence of the latter increases along with the destabilization of Tyr1–Asp3 interaction, illustrating gradual shifting of Asp3 engagement from the N-terminus to the turn region.

In the transition state phase (Figure 2T), the N-terminal hydrogen bond network, which is observed in the U–P states, becomes entirely absent, liberating the Asp3 side chain, which now repositions and starts maintaining key hydrogen bonds through its carboxyl group with residues 5–7. These interactions take over the role in the stabilization of the turn region from previously mentioned Pro4 and Gly7 which now breaks due to main chain reorientation. In this phase, also both hairpin arms start to interact through main chain hydrogen bonds between Asp3 and Thr8, as well as yet transient hydrogen bonds that connect the N-terminal Tyr1 to the C-terminal Tyr10, and are accompanied by a hydrophobic contact between Tyr2 and Trp9 side chains.

In the folded phases (Figure 2F1,F) the aforementioned interactions solidify and fix hairpin arms in their native configuration. The transition between close to folded, F1, and fully native, F, structures is related to the permanent stabilization of the hydrophobic interaction formed by Tyr2 and Trp9 and rotation of the Tyr1 phenol ring such that it covers hairpin termini, thus sealing the terminal salt bridge through its dehydration.

### 3.3 Granger Causality for CLN025

**Figure 4:**
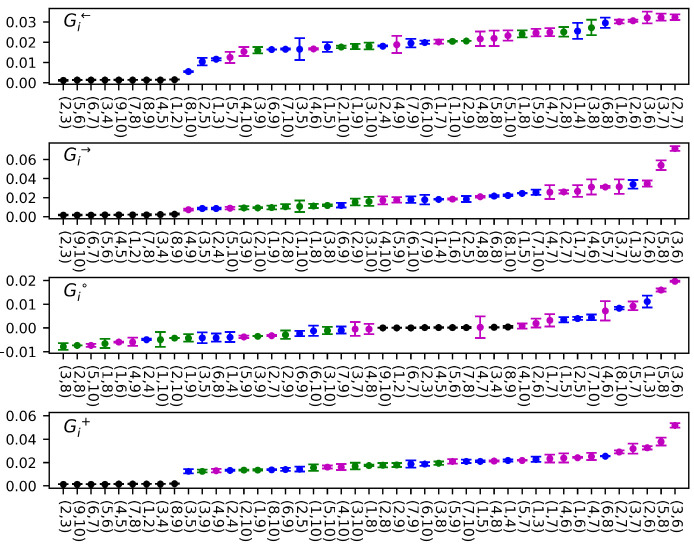
Contact-based descriptors of Granger causality. Color codes for contact groups: magenta, turn; blue, arms; green, ladder; black, direct.

The values of descriptors calculated for individual inter-residue contacts based on the complete Granger causality matrix, **J** (Supporting Information), are presented in Figure 4.

In general, the obtained amplitudes are rather low, indicating only subtle G-causal relations within the peptide. Nevertheless, independent results based on the first half and the second half of the trajectory (error bars in Figure 4) appear to be consistent, suggesting that the analysis has indeed converged. Notably, all contacts between directly neighboring residues (Figure 4, black) invariably receive 0 scores, as should be expected based on the independence of their contact distances determined by covalent bonds from peptide conformations. In order to further validate the significance of the results, we repeated all calculations for a trajectory in which frames were randomly shuffled, thus destroying all existing correlations. Having checked that the distribution of such determined *G* values in their respective categories is Gaussian, we used the Student’s *t* test to estimate the probability of a null hypothesis that the true results belong to the same ensemble (see the Supporting Information for details). Such obtained *p* values turned out to be significantly lower than 0.01 in the case of all contacts in all categories, except for contacts between neighboring residues.

An upper range of the *G*^→^ and *G*^←^ parameter spectrum is dominated by contacts belonging to the hairpin turn region (Figure 4, magenta). It is consequently reflected by their dominant *G*^+^ predictability indicator values, manifesting the highest overall involvement in G-causal relations. The above findings imply that rearrangements within this region foreshadow downstream conformational changes (high *G*^→^) and also that they are a culmination of preceding steps (high *G*^←^). Taken together, this suggests that turn rearrangements may play a major role during the transition state, constituting the threshold between folded and unfolded states. Indeed, one of the highest scoring contacts, Asp3–Thr6, which is (un)formed during the transition phase (Figure 2), has been identified to play a major role in the turn nucleation and stabilization in our analysis (section 3.2) as well as other^19,31^ analyses of CLN025 folding.

On the contrary, ladderlike contacts between opposite hairpin arms (Figure 4, green) are characterized by overall much lower *G*^+^ values. In particular, their *G*^→^ indices are low, implying that on the basis of events occurring within this group of contacts little can be predicted concerning general peptide dynamics. This leads to a conclusion that neither unfolding nor folding processes proceed as the sole result of fluctuations in ladderlike interactions until changes within the turn region take place. Notably, Tyr1–Tyr10 and Tyr2–Trp9 contacts, considered in the context of chignolin and CLN025 folding as a proxy for the hydrophobic core,^27,28,31−33^ do not achieve distinct scores in any category. Thus, in addition to relatively high predictability indices, in particular *G*^→^ for contacts in the turn region described above, our analysis clearly supports the zipperlike rather than the hydrophobic collapse driven mechanism of CLN025 folding, at least based on the simulation under study.

A parameter that is meant to distinguish between contacts that are rather the source of G-causal relations (i.e., their time evolution carries information useful in the prediction of other contact behavior) from those that are predominantly a sink of such relations (i.e., their behavior can be predicted based on the evolution of other contacts) is *G*° (section 2.4), which adopts positive values in the former case and negative values in the latter case.

**Figure 5:**
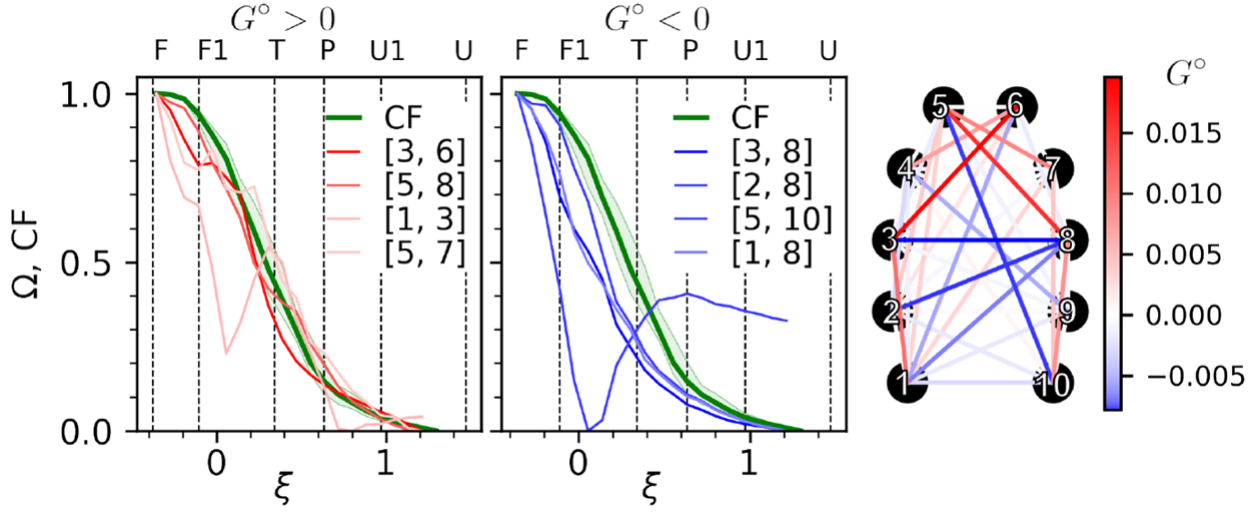
Degree of distance overlap with the native ensemble, Ω(ξ), for four contacts with highest (left plot) and lowest (right plot) *G*° values. CF, commitor function. Hairpin scheme: *G*° values for all contacts.

It seems reasonable that structural changes followed by most distinct downstream effects should take place while the system is crossing a transition state barrier since their occurrence is expected to trigger downhill evolution along the free energy gradient, typically of higher magnitude than fluctuations within any stable state. Indeed, if the state of four contacts with the highest *G*° values is followed as a function of the folding reaction coordinate (Figure 5, left plot), it can be observed that most significant changes on their route from the U state to the F state (measured as a change in the degree of overlap, Ω) with the folded state ensemble occur in the T state, closely following the CF. Furthermore, the degree of similarity of Ω(RMSD) to CF(RMSD) apparently correlates positively with the *G*° value. In contrast, major changes within four contacts at the negative end of the *G*° spectrum are consistently shifted with respect to CF toward the folded state (Figure 5, right plot).

Three out of five contacts with the highest *G*° values, including the already mentioned Asp3–Thr6 interaction, are located within the hairpin turn (Figure 5), again highlighting the importance of this region in simulated CLN025 folding under study. Notably, contacts of Glu5 with both Gly7 and Thr8 residues are involved in non-native turn stabilization (section 3.2) and need to break before the turn properly centers at Glu5. Similarly, a highly scored Tyr1–Asp3 interaction is involved in a hydrogen bond network within the N-terminal hairpin arm observed in the U, U1, P, and T states, and its vanishing enables Asp3 repositioning that is necessary for turn nucleation. These observations underscore the fact that Granger analysis is sensitive just to changes in signal components and, being also agnostic to the actual direction of the (un)folding process, does not distinguish between contact making and contact breaking.

The group of contacts with *G*° < 0 is clearly dominated by residue pairs that form parallel interactions between opposite hairpin arms, including the components of the hydrophobic core (Figure 5). This suggests that the formation of this structure completes rather than initiates folding and that its fluctuations in the folded state, e.g., temporal disruption, do not imply the commencement of the unfolding process.

There remains a question to what extent the above characterization of the folding process depends on the choice of the particular reaction coordinate. To this end we analyzed a set of six representative structures obtained for the worst (i.e., having the lowest maximum *p*(TP|ξ) value) of the considered trial reaction coordinates, which was based on the RMSD with respect to the native hairpin geometry. We found that it captures essentially the same sequence of structural rearrangements as the original one (see Figures S2 and S8), providing for the same interpretation of causal relations.

## 4 Conclusions

Temporal relations in complex biomolecules are inherently difficult to capture and to express in a quantitative manner. Our application of Granger causality analysis to chignolin allowed ranking its structural elements according to their contributions to the (un)folding process. We found that most determinant for chignolin dynamics are residues that form the β hairpin turn. Their high scores in descriptors that express involvement in causal relations are reflected in the independent observation that the major part of their transformation between folded and unfolded states occurs when the system traverses the transition state barrier. In contrast, the dynamics of contacts that are formed between hairpin arms, including those contributing to the peptide’s hydrophobic core, turn out to encode comparably little information concerning the time evolution of the system. Taken together, these results support the conclusion that the molecular dynamics trajectory under study depicts chignolin folding in agreement with a zipperlike rather than a hydrophobic collapse mechanism.

The above findings indicate the potential of Granger causality analysis to provide objective measures useful in the interpretation of biomolecular dynamics in the context of already existing hypotheses, such as the debated mechanism of β hairpin folding. Notably, however, the method is not dependent on prior knowledge or assumptions concerning the system of interest that in the case of typical MD analysis are necessary to devise suitable descriptors. This gives a possibility for obtaining insights that otherwise might be left unnoticed, such as the apparently important role of contacts within the N-terminal hairpin arm, whose breaking proved to be necessary to initiate the repositioning of the hairpin turn and subsequent folding.

A limitation of the considered approach is the need for long MD trajectories that contain multiple realizations of the process under study. Obtaining sufficient sampling for more complex systems than the one considered here will remain challenging. In this respect, an interesting route may involve the adaptation of multiple, short runs in conjunction with Markov state models to provide proper weighting of individual transitions between metastable system states. Another possible improvement may be based on the replacement of the autoregressive model used to determine the Granger causality matrix with a more powerful approach. Aside from already mentioned entropy transfer, machine learning based forecasting methods, which are constantly gaining advantage over classic statistical approaches,^65^ may be considered to increase the sensitivity of the causality analysis.

## Supporting Information Available

Derivation of Yule–Walker method for MVAR parametrization; reaction coordinate selection and validation; contact formation and turn location during CLN025 folding; Granger causality matrix; statistical validation of contact-based causality descriptors (PDF)
