# Supplementary material for: Granger Causality Analysis of Chignolin Folding
Source: J Chem Theory Comput. 2022 Feb 15;18(3):1936–44. doi: 10.1021/acs.jctc.1c00945 (PMC8908741; doi:10.1021/acs.jctc.1c00945)
Supplement: Supplementary file 1 — ct1c00945_si_001.pdf [file ct1c00945_si_001.pdf]

Granger causality analysis of chignolin folding Supporting Information

Marcin Sobieraj[†,‡] and Piotr Setny[*,‡]

†*Faculty of Physics, University of Warsaw, Pasteura 5, 02-093 Warsaw, Poland*

‡*Centre of New Technologies, University of Warsaw, Banacha 2c, 02-097 Warsaw, Poland*

E-mail: p.setny@cent.uw.edu.pl

1 Youle-Walker method for Multivariate Autoregressive Model (MVAR) parameterization

Our analysis assumes that the process under study is represented by multidimensional time series, $\mathbf{x}(t) = \{x_1(t), ..., x_K(t)\}$, propagated with a time step Δt, that fulfills the condition of being mean-ergodic and cross-covariance ergodic.[1] This implies the existence of the first and the second mixed statistical moments defined as:

$$\langle \mathbf{x}(t) \rangle = \lim_{T\to\infty} \frac{1}{T} \sum_{t=1}^{T} \mathbf{x}(t), \tag{1}$$

$$\mathbf{\Gamma}(r) = \langle \mathbf{x}(t)\, \mathbf{x}^T(t - r\Delta t) \rangle. \tag{2}$$

Furthermore, it holds that $\mathbf{\Gamma}(r) = \mathbf{\Gamma}^T(-r)$, since:

$$\mathbf{\Gamma}(r) = \lim_{T\to\infty} \frac{1}{T} \sum_{t=1}^{T} \mathbf{x}(t+r)\, \mathbf{x}^T(t) = \lim_{T\to\infty} \frac{1}{T} \sum_{t'=r+1}^{T} \mathbf{x}(t'-r)\, \mathbf{x}^T(t') = \mathbf{\Gamma}^T(-r). \tag{3}$$

Based on these assumptions one can postulate an MVAR model[2] of order P, in the following form:

$$\mathbf{x}(t) = \sum_{p=1}^{P} \mathbf{A}_p \mathbf{x}(t - p\Delta t) + \mathbf{e}(t), \tag{4}$$

where A_p are $K \times K$ real matrices and $\mathbf{e}(t) = \{e_1(t), ..., e_K(t)\}$ is the white noise vector characterized by a covariance matrix $\tilde{\mathbf{V}} := \langle \mathbf{e}(t)\mathbf{e}^T(t)\rangle$. Once parameterized, the model can be applied to predict signal value at time t based on P previous steps of the signal under study:

$$\mathbf{x}_{pred}(t) = \sum_{p=1}^{P} \mathbf{A}_p \mathbf{x}(t - p\Delta t). \tag{5}$$

A possible way to parameterize an MVAR model (that is, to determine the $\tilde{\mathbf{V}}$ and a set of $\{\mathbf{A}_p\}$ parameters) is provided by the Youle-Walker method,[3,4] which seeks to minimize the trace of the covariance matrix $\tilde{\mathbf{V}}$,[2] by solving the following set of $P+1$ linear equations for $r \in \{0, \dots, P\}$:

$$\mathbf{\Gamma}(r) = \sum_{p=1}^{P} \mathbf{A}_p \mathbf{\Gamma}(r - p) + \delta_{r,0}\tilde{\mathbf{V}}, \tag{6}$$

where δ is a Kronecker delta. Under the assumption that the error of prediction from Eq. 5, given by the difference between the real and the predicted signal, $\Delta\mathbf{x}(t) = \mathbf{x}(t) - \mathbf{x}_{pred}(t)$, is a white noise, covariance matrices of $\Delta\mathbf{x}(t)$ and $\mathbf{e}(t)$ are equal: $\mathbf{V} := \langle \Delta\mathbf{x}(t)\Delta\mathbf{x}(t)^T\rangle = \tilde{\mathbf{V}}$, which implies that diagonal elements of the covariance matrix $\mathbf{V}$ can be used as quality indicators for model predictions for respective signal channels.

Below, we provide a brief derivation of Eq. 6. Starting from Eq. 4 and multiplying it by $\mathbf{x}^T(t - r\Delta t)$, we get:

$$\mathbf{x}(t)\mathbf{x}^T(t - r\Delta t) = \sum_{p=1}^{P} \mathbf{A}_p \mathbf{x}(t - p\Delta t)\mathbf{x}^T(t - r\Delta t) + \mathbf{e}(t)\mathbf{x}^T(t - r\Delta t). \tag{7}$$

Than, looking at an expected value leads to:

$$\langle \mathbf{x}(t)\mathbf{x}^T(t - r\Delta t)\rangle = \sum_{p=1}^{P} \mathbf{A}_p \langle \mathbf{x}(t - p\Delta t)\mathbf{x}^T(t - r\Delta t)\rangle + \langle \mathbf{e}(t)\mathbf{x}^T(t - r\Delta t)\rangle, \tag{8}$$

which, based on the fact that $\mathbf{\Gamma}(r) = \mathbf{\Gamma}^T(-r)$ and $\mathbf{\Gamma}(m-n) = \langle \mathbf{x}(t-n\Delta t)\mathbf{x}^T(t-m\Delta t)\rangle$, can be expressed as:

$$\mathbf{\Gamma}(r) = \sum_{p=1}^{P} \mathbf{A}_p \mathbf{\Gamma}(r-p) + \langle \mathbf{e}(t)\mathbf{x}^T(t-r\Delta t)\rangle. \tag{9}$$

Given the white noise character of $\mathbf{e}(t)$, the term $\langle \mathbf{e}(t)\mathbf{x}^T(t-r\Delta t)\rangle$ vanishes for $r \neq 0$.[1] In turn, in the case of $r = 0$, once $\mathbf{x}^T(t)$ in this term is substituted according to Eq. 4, we get:

$$\langle \mathbf{e}(t)\mathbf{x}^T(t)\rangle = \sum_{p=1}^{P} \langle \mathbf{e}(t)\mathbf{x}^T(t-p\Delta t)\rangle \mathbf{A}_p^T + \langle \mathbf{e}(t)\mathbf{e}^T(t)\rangle = \langle \mathbf{e}(t)\mathbf{e}^T(t)\rangle, \tag{10}$$

which together with Eq. 9 are equivalent to Eq. 6.

In a similar way it can be demonstrated that if prediction error $\Delta\mathbf{x}(t)$ is white noise its covariance matrix, $\mathbf{V}$ is equivalent to the one introduced in the Youle-Walker method, $\tilde{\mathbf{V}}$. Considering the expected value of $\Delta\mathbf{x}(t)\mathbf{x}^T(t-r\Delta t)$, while substituting $\Delta\mathbf{x}(t) = \mathbf{x}(t) - \sum_{p=1}^{P} \mathbf{A}_p \mathbf{x}(t-p\Delta t)$ gives:

$$\langle \Delta\mathbf{x}(t)\mathbf{x}^T(t-r\Delta t)\rangle = \langle \mathbf{x}(t)\mathbf{x}^T(t-r\Delta t)\rangle - \sum_{p=1}^{P} \mathbf{A}_p \langle \mathbf{x}(t-p\Delta t)\mathbf{x}^T(t-r\Delta t)\rangle, \tag{11}$$

which leads to:

$$\langle \Delta\mathbf{x}(t)\mathbf{x}^T(t-r\Delta t)\rangle = \mathbf{\Gamma}(r) - \sum_{p=1}^{P} \mathbf{A}_p \mathbf{\Gamma}(r-p). \tag{12}$$

Leaving the only non-vanishing term, that is for $r = 0$, and using the substitution of $\mathbf{x}^T(t)$ and further steps as for obtaining Eq. 10 we get:

$$\langle \Delta\mathbf{x}(t)\Delta\mathbf{x}(t)^T\rangle = \mathbf{\Gamma}(0) - \sum_{p=1}^{P} \mathbf{A}_p \mathbf{\Gamma}(-p) = \tilde{\mathbf{V}}. \tag{13}$$

2 Reaction coordinate selection and validation

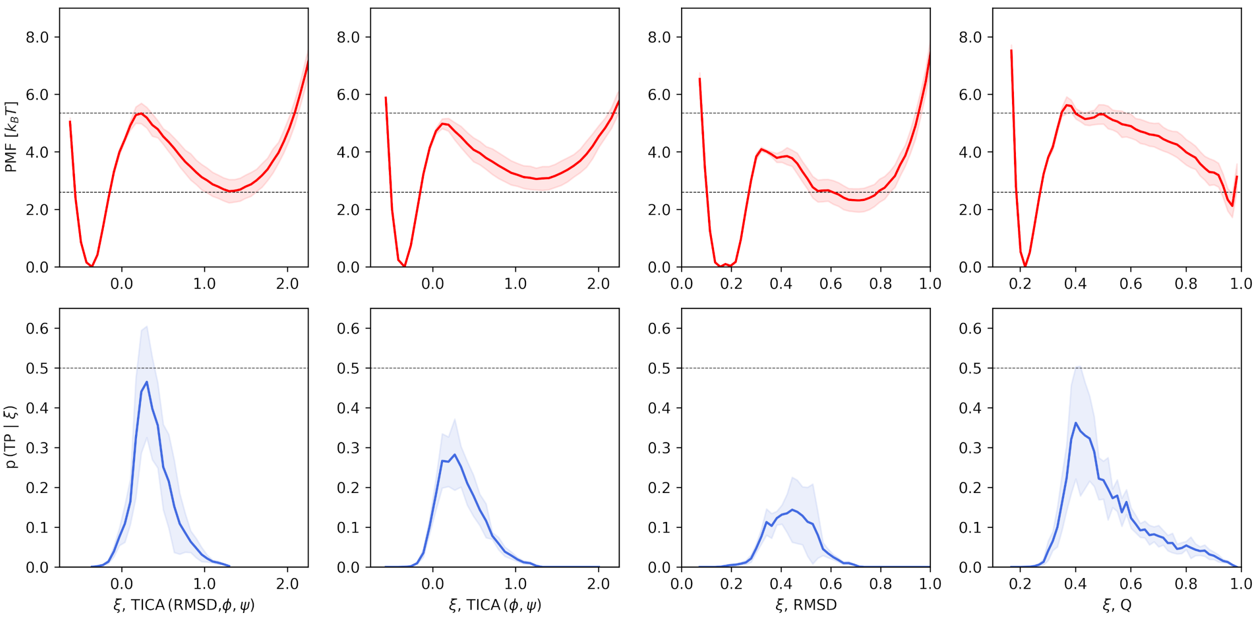

Figure S1: The comparison of considered reaction coordinates, ξ, in terms of the resulting potential of mean force, PMF(ξ), and the conditional probability of finding the system on a reactive path at a given ξ value, $p(TP\,|\,\xi)$.[5] Left to right: dominant independent component (IC) of time lagged independent component analysis (TICA) based on the combination of root mean square deviation (RMSD) from the native structure and sines and cosines of peptide (ϕ, ψ) angles; the same, but without RMSD; RMSD with respect to the native structure; the fraction of native contacts calculated according to Ref,[6] with parameters $\beta = 1.0$ and $\lambda = 1.4$. The optimal reaction coordinate was selected based on the proximity of $p(TP\,|\,\xi)$ maximum to the theoretical limit of 0.5.[5]

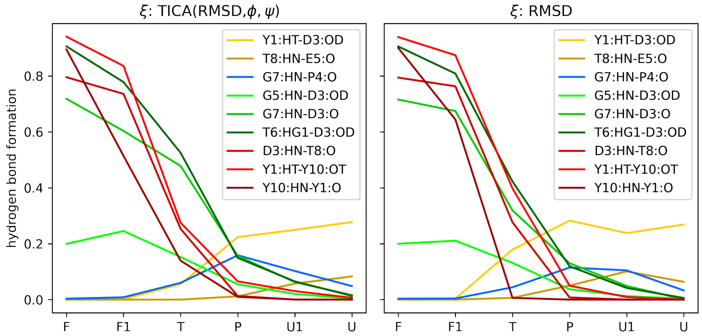

Figure S2: The comparison of major hydrogen bonds formation along the chosen reaction coordinate, ξ :TICA(RMSD,ϕ, ψ), and the reaction coordinate based on RMSD only, ξ :RMSD. Both reaction coordinates similarly capture major rearrangements in the system as described in the manuscript: gradual vanishing of the N-terminal structure and turn repositioning during folding (yellow lines), temporary formation of PRO4:GLY7 interaction (blue line), turn nucleation (green lines), and hairpin arms stabilisation (red lines). TICA-based reaction coordinate, which achieves considerably higher maximum $p(TP\,|\,\xi)$ value, resolves better the shift between turn formation and arms stabilisation around the transition state, providing more clear interpretation of the Granger causality analysis results.

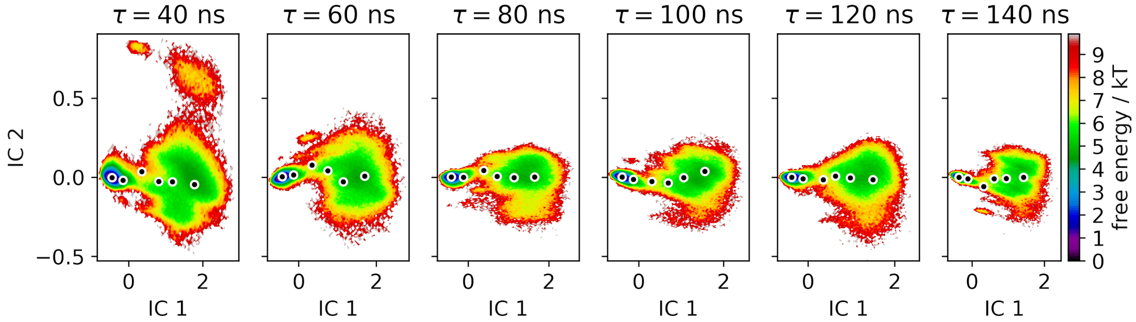

Figure S3: Free energy maps as a function of two dominant ICs for TICA solutions with increasing lag time, τ. Optimal lag time was chosen as 120 ns, based on the requirement that a) it results in two clear free energy minima corresponding to folded and unfolded states, b) the lowest free energy path between them leads along the first IC (see also Fig. S4). We note, that sets of frames and representative structures (mapped with black circles) used to visualise the (un)folding process are similar for all considered lag times (see also Fig. S5).

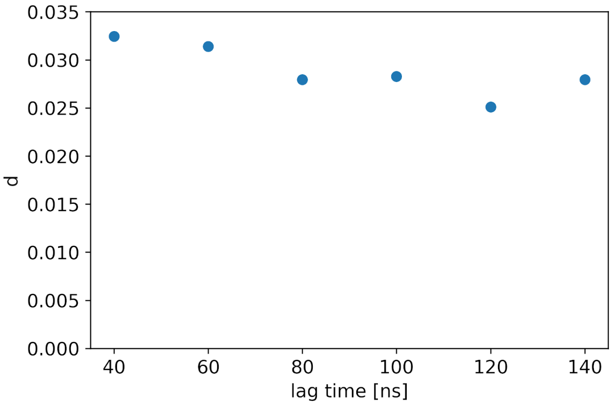

Figure S4: Root mean square deviation, d, from 0 in TICA space in all ICs $\geq$ 2, for 6 simulation frames representative for (un)folding steps obtained with different TICA lag times. d provides a heuristic measure of how well the differences between consecutive frames are explained just by the progress along the reaction coordinate (ξ = IC 1).

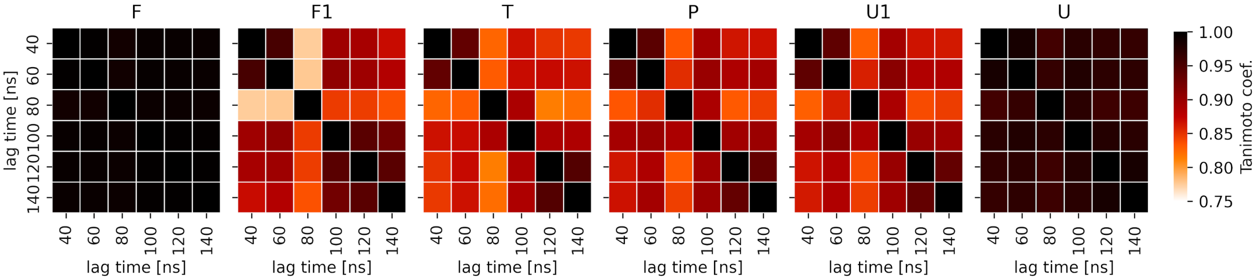

Figure S5: The overlap, measured by Tanimoto coefficient, between sets of trajectory frames assigned to consecutive folding steps based on proximity to 6 equidistant centres along the reaction coordinate, obtained for TICA analyses with increasing lag time. In most cases the overlap is > 0.85, indicating little dependence of the results on particular lag time.

3 Contacts formation and turn location during CLN025 folding

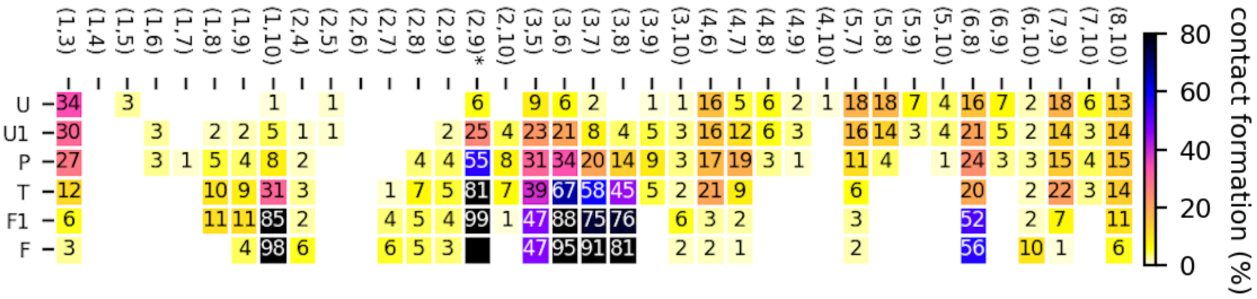

Figure S6: The frequency of contact formation in subsequent (un)folding phases based on 0.32 nm inter-residue distance cutoff. $(2, 9)^*$ denotes an additional statistics for the hydrophobic TYR2-TRP9 contact at a cutoff distance 0.5 nm.

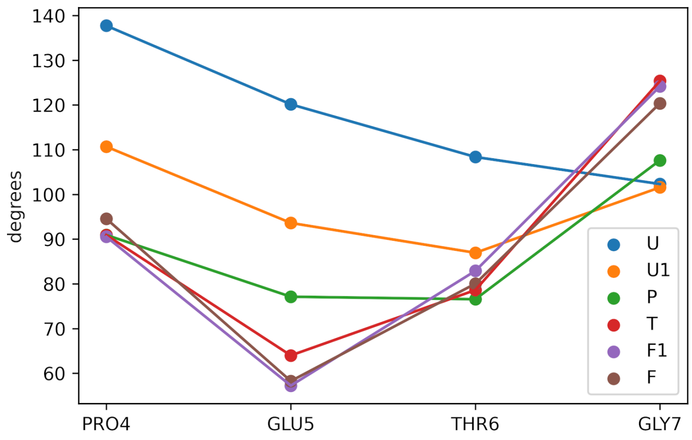

Figure S7: Medians of plane angles between Cα atoms of $i_{-2} - i - i_{+2}$ residues (reported for i-th residue) in subsequent (un)folding phases. Lines are guide for the eye.

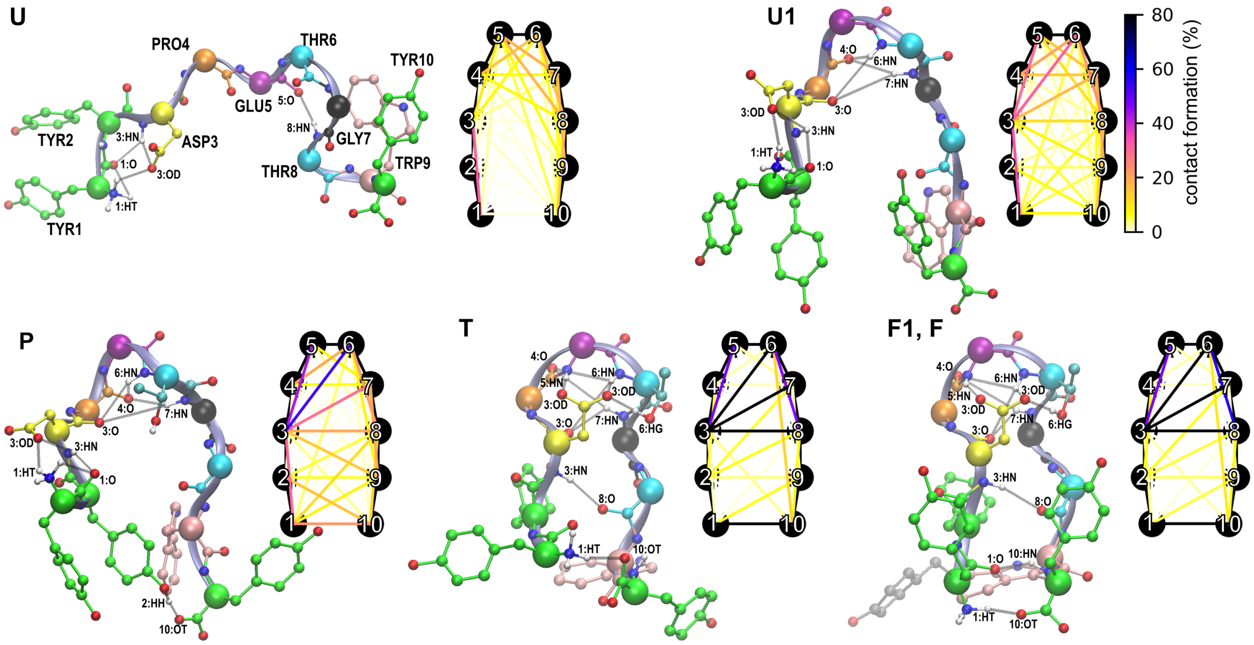

Figure S8: Representative structures for subsequent folding steps and schematic depiction of inter-residue contact frequencies obtained for RMSD-only based reaction coordinate.

4 Granger causality matrix

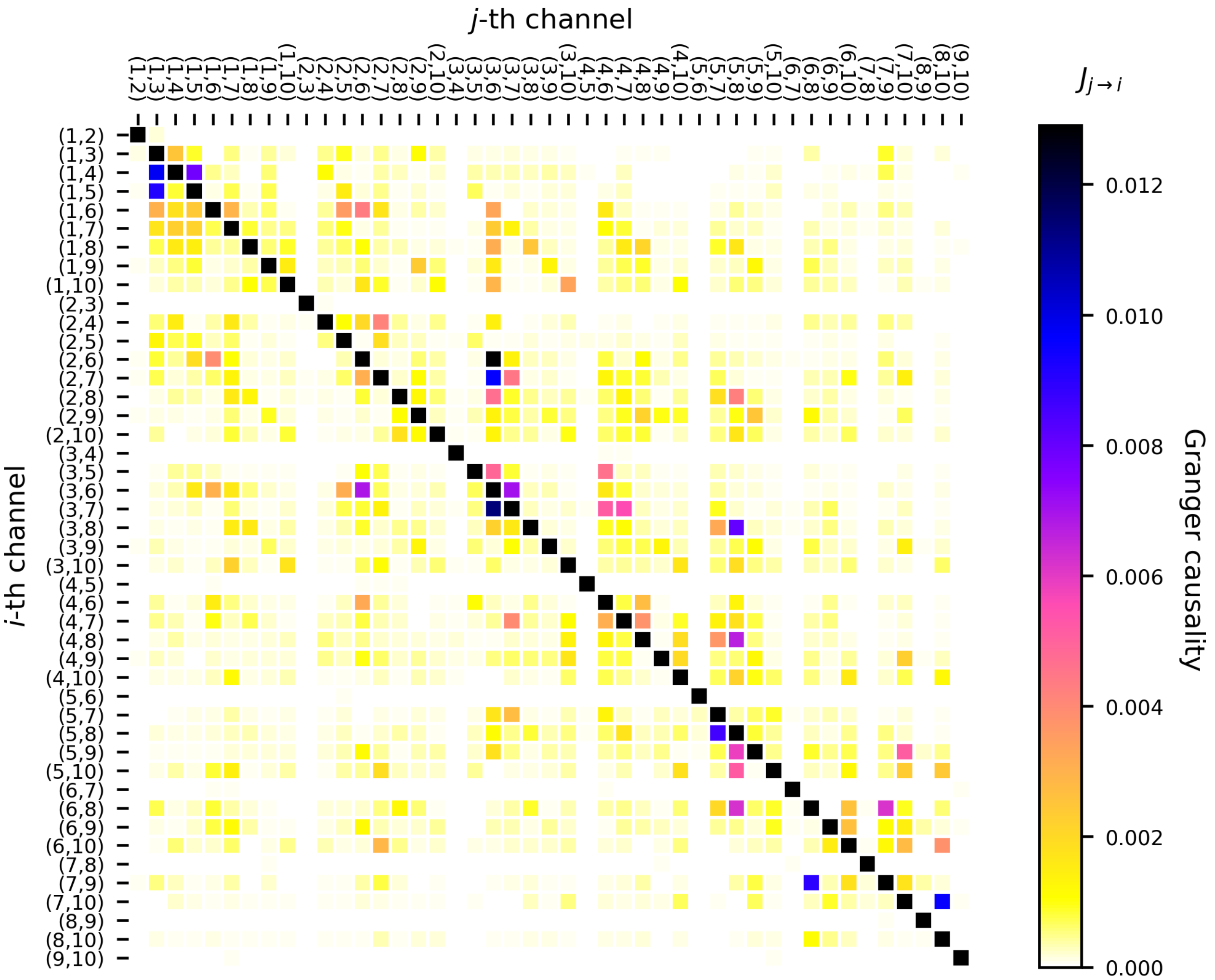

Figure S9: Full Granger causality matrix, **J**, for all inter-residue distances in CLN025.

5 Statistical validation of contact-based causality descriptors

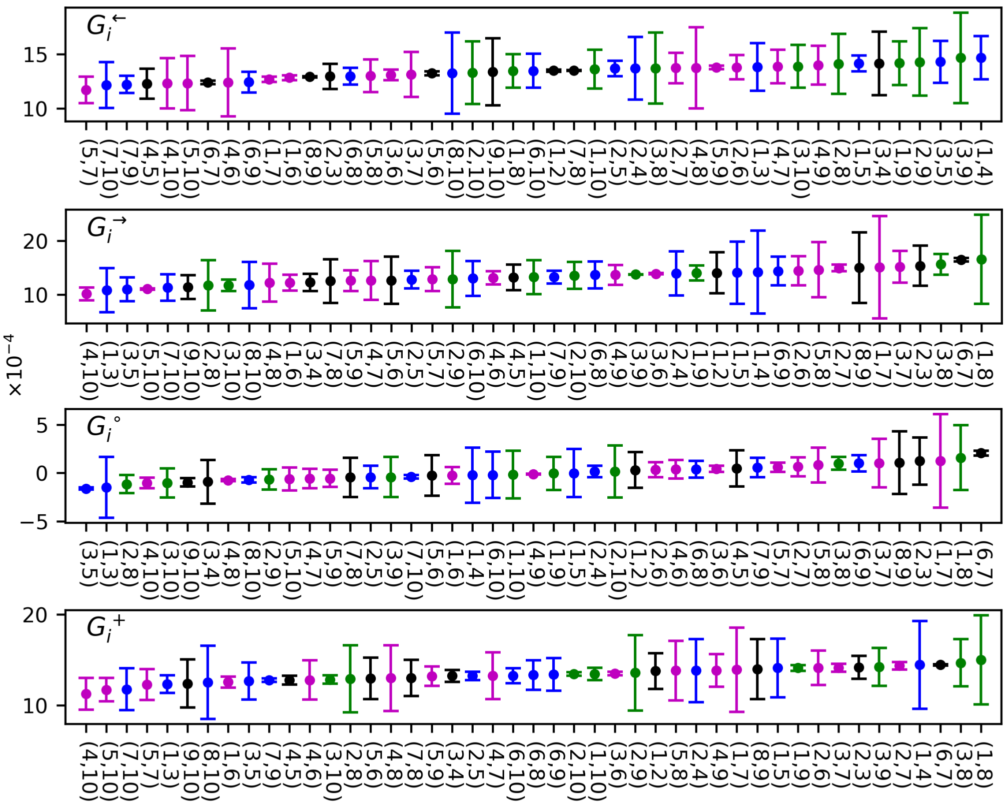

Figure S10: Contact-based descriptors of Granger causality obtained for the shuffled trajectory. Note that the Y axes are scaled by 10^{-4}. Color codes for contact groups: magenta - turn; blue - arms; green - ladder; black - direct.

Table S1: p-values for normality tests performed on sets of G values obtained form the shuffled trajectory.

test	$G^{\rightarrow}$	$G^{\leftarrow}$	G^{+}	G°
D'Agostino-Pearson	90	41	62	68
Shapiro-Wilk	97	30	82	89
Kolmogorov-Smirnov	100	71	93	95
Lilliefors	99	30	71	78

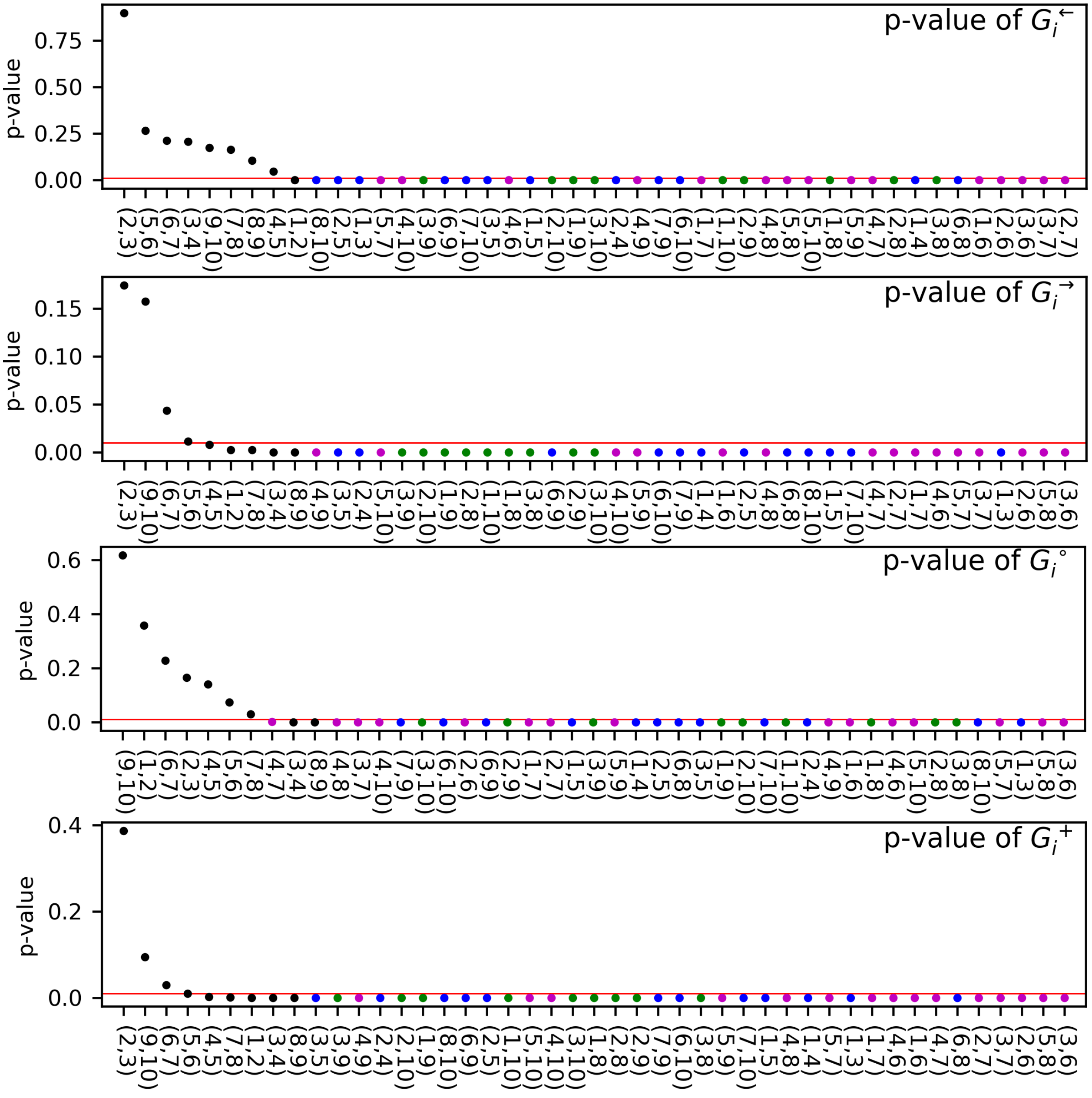

Figure S11: p-values for testing a null hypothesis that contact based descriptors of Granger causality obtained from the original trajectory belong to the same distribution as those obtained from the shuffled trajectory. Normal distributions were assumed for G values obtained upon trajectory shuffling (see above), and the Student's t-test was used to assess the p-values. Color codes for contact groups: magenta - turn; blue - arms; green - ladder; black - direct.

References

(1) Papoulis, A.; Pillai, S. U. *Probability, random variables and stochastic processes*, 4th ed.; McGraw-Hill, 2002; Chapter Spectrum estimation, pp 523–579.

(2) Ozaki, T. *Time Series Modeling of Neuroscience Data*; CRC Press, 2012.

(3) Yule, G. U. On a method of investigating periodicities disturbed series, with special reference to Wolfer's sunspot numbers. *Philos. Trans. R. Soc. A* **1927**, *226*, 267–298.

(4) Walker, G. T. On periodicity in series of related terms. *Proc. Math. Phys. Eng. Sci.* **1931**, *131*, 518–532.

(5) Best, R. B.; Hummer, G. Reaction coordinates and rates from transition paths. *Proc. Natl. Acad. Sci. U. S. A.* **2005**, *102*, 6732–6737.

(6) Best, R. B.; Hummer, G.; Eaton, W. A. Native contacts determine protein folding mechanisms in atomistic simulations. *Proc. Natl. Acad. Sci. U. S. A.* **2013**, *110*, 17874–17879.
